# Health Surveys Using Mobile Phones in Developing Countries: Automated Active Strata Monitoring and Other Statistical Considerations for Improving Precision and Reducing Biases

**DOI:** 10.2196/jmir.7329

**Published:** 2017-05-05

**Authors:** Alain Labrique, Emily Blynn, Saifuddin Ahmed, Dustin Gibson, George Pariyo, Adnan A Hyder

**Affiliations:** ^1^ Johns Hopkins Bloomberg School of Public Health Department of International Health Baltimore, MD United States; ^2^ Johns Hopkins Bloomberg School of Public Health Department of Population, Family and Reproductive Health Baltimore, MD United States

**Keywords:** surveys and questionnaires, sampling studies, mobile health, mobile phone, research methodology

## Abstract

In low- and middle-income countries (LMICs), historically, household surveys have been carried out by face-to-face interviews to collect survey data related to risk factors for noncommunicable diseases. The proliferation of mobile phone ownership and the access it provides in these countries offers a new opportunity to remotely conduct surveys with increased efficiency and reduced cost. However, the near-ubiquitous ownership of phones, high population mobility, and low cost require a re-examination of statistical recommendations for mobile phone surveys (MPS), especially when surveys are automated. As with landline surveys, random digit dialing remains the most appropriate approach to develop an ideal survey-sampling frame. Once the survey is complete, poststratification weights are generally applied to reduce estimate bias and to adjust for selectivity due to mobile ownership. Since weights increase design effects and reduce sampling efficiency, we introduce the concept of automated active strata monitoring to improve representativeness of the sample distribution to that of the source population. Although some statistical challenges remain, MPS represent a promising emerging means for population-level data collection in LMICs.

## Introduction

Since the filing of Alexander Graham Bell’s patent for the telephone in 1876, voice, and eventually, data communications networks have transformed the globe. Hard-wired landline infrastructure was a necessary developmental milestone for communities entering the modern era, rapidly connecting populations across high-income countries and most urban centers of the developing world [1]. By the 1990s, nearly every home in the United States had a fixed landline phone, which was used by national statistical agencies, like the US Centers for Disease Control (CDC) or the Census Bureau, and by polling organizations for conducting household surveys [2].

Until the first mobile phone was introduced in the early 1970s, there was no challenge to the role of the landline telephone as a tool for population-level data collection. As the global mobile phone revolution exploded in the early 2000s, a dramatic shift from landline to cellular networks began to occur. According to the CDC, by early 2005, only 7.3% of US households had shifted to mobile as their only phone connection [3]. By the end of 2015, a little over a decade since the agency began meticulously tracking household-phone ownership, only 8% of homes reported exclusive landline phone access, with 44% of homes reporting a mobile phone being their only communication access [3]. This transformation has been even more dramatic in the developing world, where landline infrastructure has been leapfrogged by the rapid deployment of mobile networks and affordable cellular telephony [1].

This transition to ubiquitous mobile phone access around the globe has had an important effect on population surveys especially in low- and middle-income countries (LMICs) where the availability of landline phone was rarely universal and surveys were conducted by face-to-face interviews (F2F). However, F2F surveys are expensive, time consuming, and often difficult to conduct in remote or conflict regions. Mobile phone surveys (MPS) are likely to reduce these challenges. In fact, several global agencies and survey firms have begun to leverage mobile phone coverage rates to collect data at random or from panels of respondents [4].

In this paper, we identified some of the key statistical considerations and challenges associated with each stage of mobile-only surveys in LMICs. We propose some novel methodological approaches for improving population representativeness and efficiency of MPS (see Table 1).

**Table 1:** Key mobile phone surveys (MPS) considerations by survey phase.

Phase	Key considerations	Mitigation
Presampling	Differences between phone owners and nonphone owners	Decreases as mobile penetration increases in LMICs; survey of nonphone owners can help understand bias
Sampling and survey execution	Source of numbers to sample from; obtaining a representative sample; multiple phone or SIM card ownership	Prescreened “valid numbers only” bank of numbers; random digit dialing; automated active strata monitoring
Postsampling	Residual differences between phone owners and nonphone owners; residual differences between respondents and nonrespondents; residual differences between single- and multiple-phone owners	Postsampling weighting

### Presampling challenges for MPS

#### Redefining the Concept of the Sampling Frame

As the level of mobile phone access and ownership reaches saturation (100%, or at least one mobile phone per eligible adult respondent) at a population level, it becomes plausible to consider the entire population of phone owners as elements of a “sampling frame” for an MPS [5]. Although some countries have reached universal level of mobile penetration, most of them have not, thus resulting in some degree of sample misalignment when comparing the theoretical sampling frame to the population at large.

**Figure 1:**
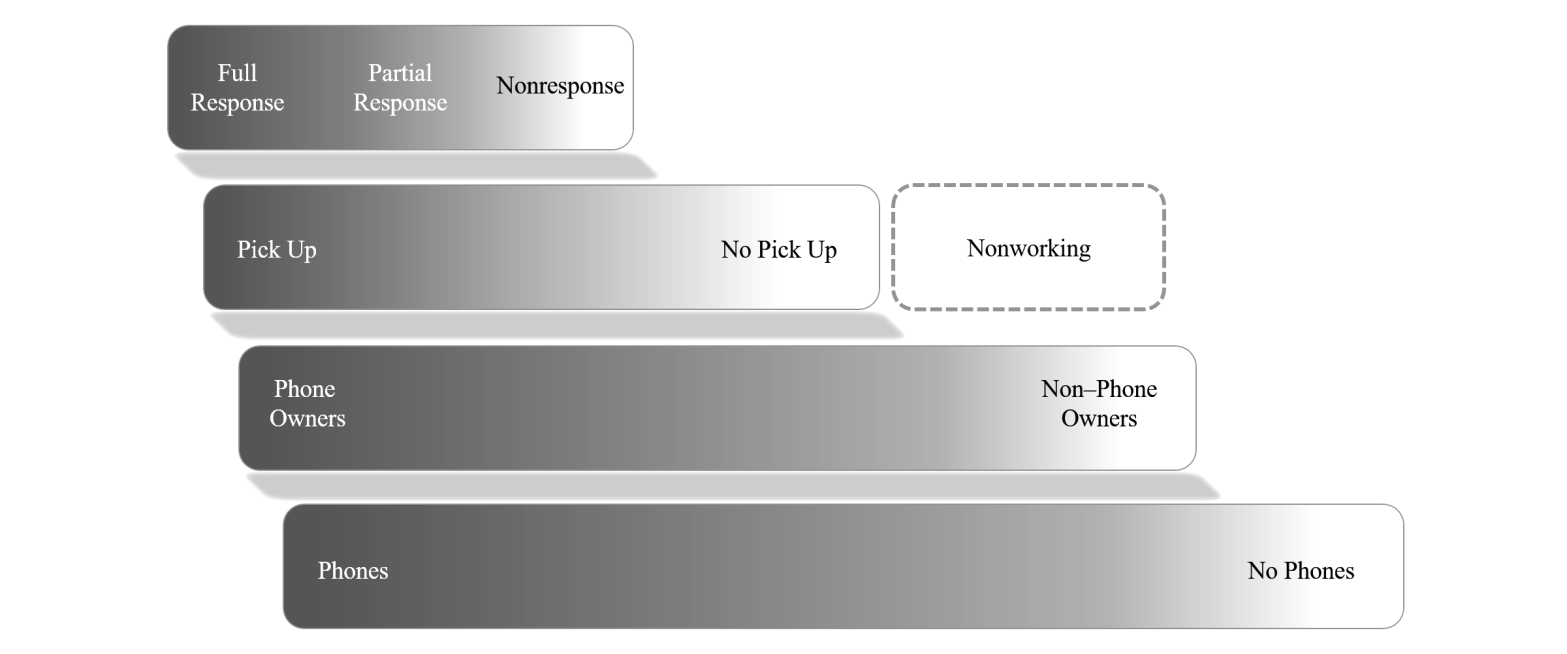
Statistical challenges for each phase of MPS. The figure illustrates the challenges in capturing specific denominators in MPS, where the bottom layer is the complete population, which comprises phone owners and non–phone owners. A cascading gradient has been used to depict the uncertainty and variability, by setting, in the proportions of either group at each layer. Various types of loss and attrition, from nonresponse to invalid numbers, reduce the total number of units sampled successfully and completely.

As shown in Figure 1 (adapted from [6]), as country-level mobile penetration and ownership increases, the amount of “white space” in the stratum, representing geographies without mobile phones, should decrease. An MPS begins with the intended target frame of the MPS as the entire population of mobile phone owners, without restriction, but still a subset of the total population until such time that a population is 100% covered (where all eligible respondents have at least 1 phone). In most cases, it is difficult to characterize the relative size and makeup of the non–phone owners, unless representative population data are available on determinants of phone ownership or surveys of characteristics of these two populations are available (or can be conducted by the researchers). It is also important to note that, as the cost of device ownership and airtime decreases, the socioeconomic composition (and associated risk factor behaviors) of phone owners and nonphone owners is also likely to change. These secular trends should be considered when comparing the results of MPS across multiple time points.

In the third layer, we see how this subset of phone owners might respond, by picking up or not, and also a new section, representing numbers that are nonexistent—an artifact of the random digit dialing process, discussed in detail below. The fourth layer depicts the possible response behaviors of the subset of those phone owners who do pick up, with the gradient representing the different possible outcomes of the respondent interaction.

#### Differences Between Individuals Who Own and Do Not Own Phones

As populations transition from either fixed or no phone access to widespread cellular networks, heterogeneity in coverage and household ownership can vary across states or districts. These differentials are likely to mirror socioeconomic and rural-urban gradients and could hinder statistically sound estimates of population characteristics and behavior if not carefully understood and managed [3]. Differences between landline and mobile phone (or combined household) respondents have been documented extensively across multiple populations over the past decade [7,8]. Many previous discussions around statistical methods and sampling strategies to mitigate the risks of bias and threats to validity have focused on high-income country settings, in the background of a strong landline or mixed-network legacy systems [9,10]. In most LMICs, landline infrastructure has remained underdeveloped or limited to larger metropolitan areas, leaving mobile phone “only” households as the predominant and more universal denominator. These mobile-only populations remain poorly characterized in most settings, with gaps in ownership/access, sometimes characterized as a facet of the “digital divide,” aligned with lower socioeconomic strata or marginalized populations [11]. In some countries of sub-Saharan Africa, where populations are sparse and distributed across a large geographic spread, extreme remoteness and cellular network unavailability may also be determinants of respondent unreachability via MPS.

### Sampling Strategies for MPS

#### Identifying a Sampling Frame and Dealing With Invalid numbers

Apart from addressing challenges in the differences between phone users and nonphone users in a population, the next important hurdle for any phone survey methodology begins with the development of a representative and valid sampling frame, broadly defined as the set of eligible numbers from which the sample of telephone households is selected [9]. The sampling frame has been a critical component of traditional surveys as a major focus of survey representativeness and costs [9]. For telephone surveys, three approaches to sampling frame development have traditionally been used such as random digit dialing (RDD), list-assisted design, and a multiple-frame sampling method that combines the two approaches.

Unlike telecom companies in many high-income countries, in LMICs, providers seldom maintain (or publish) directories of active mobile phone users and their numbers [7]. Without accurate published or network-operator provided lists to select from, RDD remains the most appropriate sampling approach. In landline surveys conducted in the United States, sampling frames are often developed by obtaining known prefixes to which random suffixes are appended. In many countries, mobile network operators (MNOs) are provided prefixes different from each other (eg, 019, 017) by the government, followed by a fixed set of numbers, which helps minimize ineligible random number combinations. As the populations using different MNOs may differ in geographical distribution and other characteristics (eg, income or education level, based on market segments targeted for low cost, entry-level prepaid plans), using known MNO prefixes also helps to ensure proportionate representation in the sample of the market share held by different operators. On the basis of this information, a large pool of possible numbers, an order of magnitude larger than the necessary sample, can be computer generated as the first “pool” of numbers to dial randomly. New pools can be generated, with care taken not to re-create numbers, if a pool is depleted. Although some network operators provide specific feedback (in the form of digital signals) when an invalid number is dialed, this practice is not universal.

Due to invalid sequences that are essentially unavoidable in an RDD, the required sample size should be inflated by dividing with a factor 1-Y, which estimates the proportion of nonworking numbers, to yield the number of random mobile phone numbers to be generated. This factor can be determined by first creating a smaller “test” pool of numbers to determine a likely proportion of real numbers through a practice RDD round. As the numbers are dialed, if a number is identified as a nonhousehold or definitively found to be nonworking on the first call, then it should be excluded and the next one be dialed. If a call is not answered, it can be redialed a predetermined number of times before it is identified as nonworking and replaced with a new number from the list.

#### Accelerated Sequential Replacement

To ensure that correct sample size is obtained, traditional landline surveys have employed a process called accelerated sequential replacement. This is an iterative approach that selects numbers from a purposefully expanded sample of random numbers and replaces those definitively identified as nonworking at the end of a given operational stage, such as the day or shift. Three statuses are usually assigned to the outcome of a call such as verified household, verified nonhousehold or nonworking, and unresolved (eg, no answer or strange noise, but not verified as nonworking). Before the beginning of the next stage, those numbers that have been verified as nonworking are replaced with an equal number of new random numbers from the randomly generated list. After a predetermined number of calls, unresolved numbers are assumed to be nonworking and are also replaced. Although traditionally this was a manual process, now it can be easily automated and invalid numbers can be replaced automatically, instead of doing in stages, thereby reducing the total time required to conduct the survey.

#### An MPS Approach to Quota-Driven Sampling: Automated Active Strata Monitoring

The relatively low cost and automated nature of most MPS technologies, combined with the vast size of the denominator being selected from—effectively every mobile phone owner in the population with an active subscription, connected to a network—allows us to consider an approach that continues to attempt to fill a particular target demographic stratum of a population until that stratum’s desired sample size is reached. In this quota-driven sampling procedure, a priori “sample size” is determined with a known statistical precision level, and the sample is selected from an RDD list through a probabilistic sampling procedure. Differences between the composition of the general population and the population of phone owners, in terms of gender, age, socioeconomic status, urban versus rural residence, and geographic origin can be mitigated through establishing target quota, based on the relative proportion of individuals of a particular combination of relevant characteristics in the population at large. Recent census data can be used to assess strata-specific population distribution, or in case where data from a recent census is not available, information from the most recent demographic and health surveys (DHS) may be used. The DHS are conducted in over 90 LMICs and provide nationally representative data on these strata-specific population distributions.

Given the digital nature of MPS, real-time data streams can be monitored, and strata actively “closed” once the required sample size for that subgroup has been met. This process can be automated or monitored by study implementers. The concept, automated active strata monitoring (AASM) also allows many more strata to be chosen to minimize the possible effects of nonprobabilistic sampling from the parent population. In this process, when a participant answers the phone, the first survey questions should establish their demographic, education, and other sociodemographic information of interest to determine stratum contribution. If the required number of targeted respondents has already been reached in their stratum, no further questions are asked, and they will be excused from completing the survey. Conversely, if more respondents are still required in their stratum, they are led through the survey questions.

**Figure 2:**
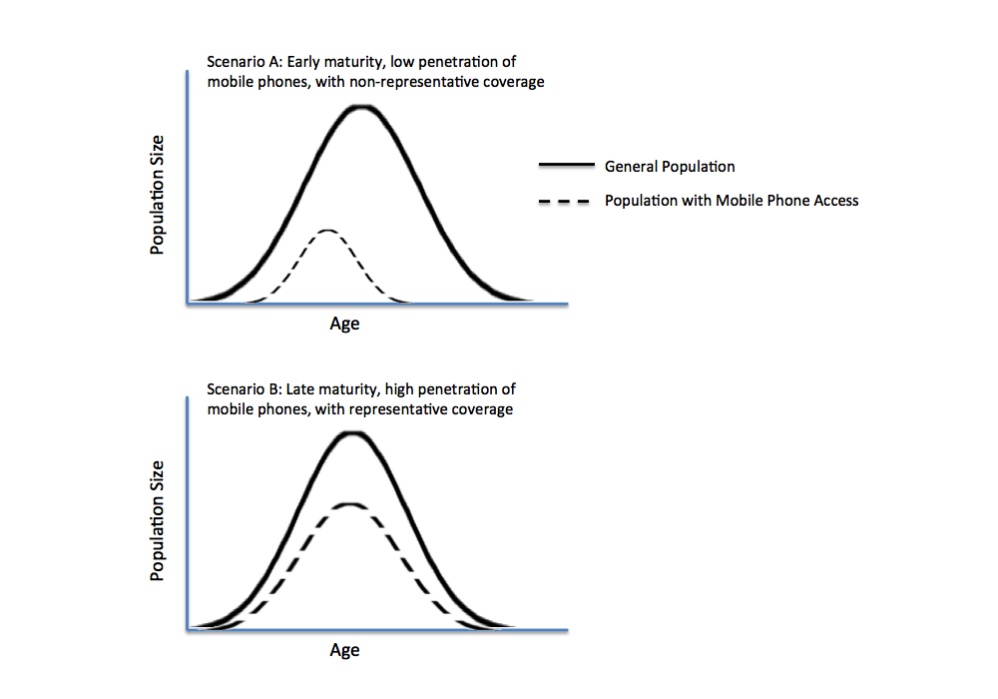
Mobile phone access across two theoretical populations, using age as an illustrative respondent characteristic through which representativeness can be assessed. The figure illustrates a hypothetical population distribution against a distribution of mobile phone ownership, under conditions of low (scenario A) and high (scenario B) mobile penetration. In scenario A, common to populations where mobile phones have recently been introduced, obtaining a representative sample through MPS may not be feasible, even using AASM. As mobile markets mature, the overlap in distributions increases, allowing methods like AASM and random digit dialing to improve the capture of a sample that closely reflects the population-at-large.

AASM is not plausible in traditional household surveys, as the marginal cost and time required to visit more households, in the effort to complete one or more unfilled strata, becomes prohibitively expensive. With the extremely low cost of MPS, in contrast to traditional F2F methods, and high levels of mobile phone coverage, for the first time in history, the survey denominator is theoretically, in many cases, the entire population (see Figure 2). For example, if the population of mobile phone owners between the ages of 60 and 70 years is a small percentage of the total population, even under less-than-complete mobile phone saturation, the denominator of phone-owning individuals in the population-at-large may number in the tens of thousands or millions. Although a greater number of calls may be required to enroll a sufficient number of these relatively “smaller” population strata, the cost and feasibility of doing so through MPS is much more reasonable. Using AASM, much more granular stratification can be achieved with MPS, ensuring greater representativeness and minimizing potential selection bias due to nonrepresentativeness in select population strata, common with small sample-frame surveys.

For traditional survey methods, an inherent disadvantage of quota-driven sampling is that it is a nonprobabilistic approach, and although certain characteristics of interest have been chosen to recreate a sample reflective of the population as a whole, other unmeasured characteristics have not been accounted for because the underlying population distribution is unknown. In a random sample, the distribution of both measured and unmeasured characteristics withstand a better chance of reflecting the actual population. With AASM in MPS, however, the population distribution strata are preserved through sampling without replacement and quota restrictions to mitigate the oversampling of certain population groups. This method does require, however, access to reliable and recent statistical information regarding the parent-population’s characteristics. These may be available from national surveys and other globally standardized surveys (eg, DHS), although recent information may not be readily available for some populations.

Given the large size of the population being sampled from, sampling without replacement from the entire population of phone owners can usually continue until the desired sample size in every sociodemographic stratum is achieved. In some cases, such as scenario B in Figure 2, stopping rules might be useful if certain age quota cannot be met, despite extended efforts, simply because those population groups cannot be reached directly using mobile phones. However, innovative methods similar to snowball sampling could be tried by requesting respondents to hand their phone to a family member, which is fulfilling specific, hard-to-obtain, requisite criteria.

### Postsampling Weighting

#### Differences Between Phone Owners and Non-owners

It is well recognized that inequity exists in mobile phone access, and phone ownership is not equally distributed across the population in many countries. Young, male urban populations are more likely to have a mobile phone compared with older, female rural populations. Consequently, a major problem of mobile survey is the selectivity bias due to ownership heterogeneity. A common method is to conduct weighed analyses with poststratification adjustments to reduce the selectivity bias and to improve population representativeness.

**Figure 3:**
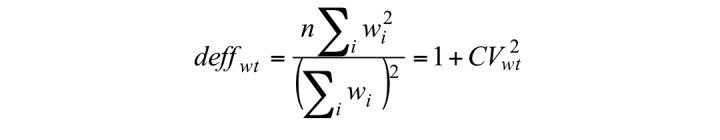
The design-effect (deffwt) of weighted estimate. Here wi is the weight (inverse of selection/participation probability) in the i-th stratum.

Poststratification with weighting, however, is not without problems. Kish has shown that the variance σ^2^/n of a weighted estimate is inflated by a factor of 1+CV^2^_wt_, where CV^2^_wt_ is the relative variance of the sampling weights [12]. The design-effect (deff_wt_) of weighted estimate is formally written as shown in Figure 3.

where *w*_i_ is the weight (inverse of selection/participation probability) in the i-th stratum As larger is the variability of the weights, the larger is the design-effect, which reduces the efficiency of the sampling design (but increases variance with larger confidence intervals and reduces the ability to reject null hypothesis). A larger deff >1 reflects the loss of precision due to effective reduction in sample size.

A multicountry study in Afghanistan, Ethiopia, Mozambique, and Zimbabwe by the World Bank shows that the design-effects due to weighting were quite large and are 6.3, 11.6, 5.2 and 1.8, respectively [5].

Trimming extreme weights is often suggested to reduce the coefficient of variation (CV) of weight, which may bias the results. We propose, for MPS, reducing the variability in weighting by restricting quota of interviews for each strata to the original sample allocation size. An AASM approach is expected to substantially reduce the CV of weights and thus the *deff* impact of poststratification weighting.

#### Minimizing the Effects of Nonresponse and Incomplete Surveys

Previous research indicates that dropout rates might be higher with MPS as people are more likely to be occupied than when contacted in person or on a landline and be less available to complete the full survey [7]. Battery or connectivity/network failure during the survey is also a factor unique to mobile surveys that could contribute to dropouts [7]. However, the response rate could also potentially be higher for MPS than for landline surveys, as the available period to reach respondents is wider, instead of being limited to evenings and weekends when an individual is at home similar to the case with landline surveys.

**Figure 4:**
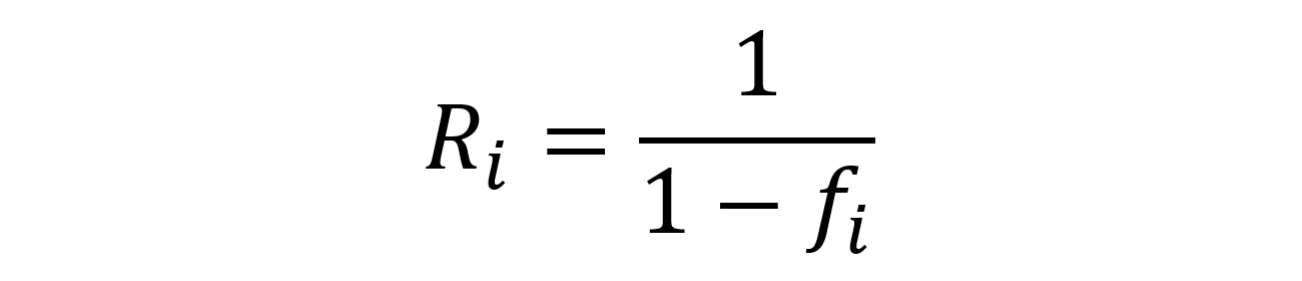
Nonresponse weighting, using a factor, Ri, where f is the nonresponse rate for the i-th group.

To minimize the effect on the data from dropouts that do occur, the order of the survey modules could be randomized so that each module has the opportunity to be placed at the beginning of an interview to ensure a sufficient number of responses to each set of questions. Additionally, to keep mobile phone interviews short, questions asked should be limited to “important” key indicators identified in consultation with country policy makers and stakeholders, as well as the literature. Further, to control for the differences between those who respond to the survey and those who refuse, during the survey the number of those who choose not to respond should be recorded. The results should be adjusted postsurvey by nonresponse weighting, using a factor, *R*_i_, expressed formally in Figure 4.

#### Multiple Users per Phone

In low socioeconomic populations, mobile phones may be shared among members of a household or among neighbors, which may lead some individuals to be less likely to be surveyed [11,13]. To mitigate this risk, MPS implementations can randomize the time of day when calls are made, to reach individuals who typically use the phone at different times during the day, or purposefully target times of day when members of the family are more likely to be reached (eg, in the evening, when the phone owner returns from work outside the home). AASM, described above, will also aid in ensuring that a sufficient number of those with less frequent use of the phone, for example, women and older individuals, have increased chances of being included in the survey.

#### Multiple Phones per Person or Household

A concern commonly voiced when considering populations where cellular phone use is high is that people with more than 1 mobile phone have a statistically higher probability of selection from a population, with the relative probability of being selected proportional to the number of phones owned by that user. Although statistically correct, the practical implications of this relatively rare situation, from a population perspective, are negligible. Using RDD in a large population (eg, the denominator of every possible phone number in a country), the individual probability of selection of an individual is very small, even if the user has more than 1 phone.

The selection probability of an individual person can be calculated as follows: (n, sample size targeted × N[p], of phones per person)/(N[T], total number of phones). When the N(p) in numerator is 1, individuals with one phone have the equal selection probability (approximately) to simple random sampling. If N(p) = 2, the selection probability is doubled and so on thus increasing the selection probability proportional to the number of phones a person may have.

Table 2 below illustrates the individual probability of selection, as the proportion of the population with more than one mobile phone increases. As illustrated, in a population of 100 million phone owners, even if 10% of the population owns 3 mobile phones, the probability that one of these individuals is selected is still 2.5 × e^-8^. This probability decreases as the proportion with more than 1 mobile phone increases. Although the theoretical probability of inclusion of those with multiple phones is three times that of someone with only one phone, in practice, the overall likelihood of contacting each participant remains infinitesimally low. Nonetheless, given that individuals with multiple phones are likely different from those with only one phone, negatively weighting responses from those individuals, or otherwise treating their contributed data as different from the majority of respondents may be worth considering. Over the course of our planned work, we aim to further explore this important issue to better elucidate the impact that multiple-phone ownership might have on the data collected in MPS.

**Table 2:** Illustration of the extremely low individual probability of inclusion of those with 1 phone and those with 3 phones in a theoretical population of 100 million and how these probabilities change as the proportion with multiple phones increases.

One mobile phone			Three mobile phones			Relative probability of inclusion (g) (f)/(c)
Percentage of 100 million population with one mobile phone (a)	Number of mobile phones in group (b) 100,000,000 × (a)	Individual probability of inclusion (c) 1/([b]+[e])	Percentage of 100 million population with 3 mobile phones (d)	Number of mobile phones in group (e) 100,000,000×(d)×3	Individual probability of inclusion (f) 3/([b]+[e])	
100%	100,000,000	1.00E-08	0%	0	3.00E-08	3
90%	90,000,000	8.33E-09	10%	30,000,000	2.5E-08	3
80%	80,000,000	7.14E-09	20%	60,000,000	2.14E-08	3
70%	70,000,000	6.25E-09	30%	90,000,000	1.88E-08	3
60%	60,000,000	5.56E-09	40%	120,000,000	1.67E-08	3
50%	50,000,000	5E-09	50%	150,000,000	1.5E-08	3

In countries where a significant proportion of the population has more than one mobile phone, the selection probability could be adjusted to take this into account. This has been done in landline surveys using an adjustment factor A, expressed formally as:

A=1/T_i_

where T_i_ is the number of phones of the *i*-th person [14]. The inverse probability of selection multiplied by A yields the adjusted weight of each respondent in the survey [14].

### Unresolved Statistical Challenges for MPS

#### Geographic Stratification and Representation

Determining the geographic location of respondents is a challenge unique to mobile surveys that is uncommon for landline or household surveys, where the area code or zip code of a respondent tends to be known. As such, if geographic balance is sought, screening questions may be necessary to associate the mobile phone respondent with a geographic location [7]. In population where mobility levels are high, questions should be developed a priori to satisfactorily assign geolocation (eg, where they spend the greatest number of days in a typical week or month) or to be determined by the participant themselves, directly stating a district or region of the country in which they reside.

#### Cost and Time Required to Obtain a Clean Sample (Removing Nonvalid Numbers)

With all RDD surveys, as phone numbers in the sample are randomly generated, it takes time to exclude nonworking numbers and eventually obtain the necessary sample size. This issue is especially pronounced for mobile surveys. In one example, it took almost 30 hours to remove 6872 nonworking mobile numbers compared with a landline sample, which took only 4.5 hours [7]. There are services that offer verification of phone number samples, but, as mentioned earlier, the associated costs may exceed the cost of the nonproductive call itself. Some active numbers could be also be flagged as nonworking in error [15]. Automated processes expedite the exclusion of nonworking numbers and replacing them, without requiring surveyor time. Notwithstanding, the time and cost necessary to isolate working numbers is still extremely low, when compared with the resources required to perform F2F household surveys. One study found that mobile interviews saved US $14 per participant compared to F2F interviews and took much less time because transportation to the field was not required [16].

### Conclusions

The mobile phone revolution has presented an unprecedented opportunity to collect public health-related data directly from populations. Near-universal connectivity has created massive population denominators, which are accessible to researchers interested in supplementing traditional F2F methods with data from MPS. Leveraging large, connected populations for high-quality survey research requires careful consideration of both unique and shared challenges across traditional F2F, landline, mixed population, and mobile-only surveys.

The low cost and automated process of deploying MPS allows for innovative approaches such as AASM to be used to create samples that reflect the population-at-large, acknowledging that nonprobabilistic methods may be accompanied by unmeasurable biases. It is important that researchers working on using MPS methods consider these and, where possible, try to collect data which will improve not only the quality of the study but also our understanding of the strengths and limitations of this method.

We face a unique reality in a growing number of countries, where approaches like RDD now allow virtually every member of a population to be reached and surveyed about important public health issues. In most high-income countries, the over use of mobile networks by telemarketers has reduced our capacity to take advantage of these methods. Robust methods in sampling and design will help maximize the value of MPS data in these countries as a useful approach to population surveillance.

## Abbreviations

AASM: automated active strata monitoring
CV: Coefficient of variation
F2F: face-to-face
LMICs: low- and middle-income countries
MPS: mobile phone surveys
RDD: rapid digital dialing
MNO: mobile network operator
DHS: demographic and health surveys
